# In Vivo Evaluation of Gallium-68-Labeled IRDye800CW as a Necrosis Avid Contrast Agent in Solid Tumors

**DOI:** 10.1155/2021/2853522

**Published:** 2021-12-13

**Authors:** Marcus C. M. Stroet, Erik de Blois, Joost Haeck, Yann Seimbille, Laura Mezzanotte, Marion de Jong, Clemens W. G. M. Löwik, Kranthi M. Panth

**Affiliations:** ^1^Erasmus MC, University Medical Center Rotterdam, Department of Radiology & Nuclear Medicine, Rotterdam, Netherlands; ^2^Erasmus MC, University Medical Center Rotterdam, Department of Molecular Genetics, Rotterdam, Netherlands; ^3^AMIE Core Facility, Erasmus MC, Rotterdam, Netherlands; ^4^Life Sciences Division, TRIUMF, Vancouver, Canada; ^5^CHUV Department of Oncology, University of Lausanne, Lausanne, Switzerland

## Abstract

Necrosis only occurs in pathological situations and is directly related to disease severity and, therefore, is an important biomarker. Tumor necrosis occurs in most solid tumors due to improperly functioning blood vessels that cannot keep up with the rapid growth, especially in aggressively growing tumors. The amount of necrosis per tumor volume is often correlated to rapid tumor proliferation and can be used as a diagnostic tool. Furthermore, efficient therapy against solid tumors will directly or indirectly lead to necrotic tumor cells, and detection of increased tumor necrosis can be an early marker for therapy efficacy. We propose the application of necrosis avid contrast agents to detect therapy-induced tumor necrosis. Herein, we advance gallium-68-labeled IRDye800CW, a near-infrared fluorescent dye that exhibits excellent necrosis avidity, as a potential PET tracer for in vivo imaging of tumor necrosis. We developed a reliable labeling procedure to prepare [^68^Ga]Ga-DOTA-PEG_4_-IRDye800CW ([^68^Ga]Ga-1) with a radiochemical purity of >96% (radio-HPLC). The prominent dead cell binding of fluorescence and radioactivity from [^68^Ga]Ga-1 was confirmed with dead and alive cultured 4T1-Luc2 cells. [^68^Ga]Ga-1 was injected in 4T1-Luc2 tumor-bearing mice, and specific fluorescence and PET signal were observed in the spontaneously developing tumor necrosis. The ip injection of D-luciferin enabled simultaneous bioluminescence imaging of the viable tumor regions. Tumor necrosis binding was confirmed ex vivo by colocalization of fluorescence uptake with TUNEL dead cell staining and radioactivity uptake in dichotomized tumors and frozen tumor sections. Our presented study shows that [^68^Ga]Ga-1 is a promising PET tracer for the detection of tumor necrosis.

## 1. Introduction 

Excessive occurrence of cell death is a hallmark for severe disease in many pathologies, such as sepsis [1], pancreatitis [2], or acute myocardial infarction [3]. Moreover, most solid tumors develop necrotic tissue due to the growth rate of the tumor mass surpassing the rate of vascularization. As a result, tumor necrosis is often associated with aggressive tumor types and poor disease prognosis [4, 5]. Currently, noninvasive techniques for necrosis detection are lacking in the clinic. There have been several agents reaching clinical trials targeting apoptosis markers, for instance, radiolabeled Annexin V, which targets exposed phosphoserines [6], or [^18^F]ICMT-11 targeting caspase-3/7 activation [7, 8]. However, due to the lack of specificity, these tracers have so far failed to reach the clinic [9–11].

We and others have previously explored the possibility of using necrosis avid contrast agents (NACAs) for noninvasive imaging of cell death in tumors [10, 12]. There are several promising classes of NACAs mentioned in the literature such as Rhein and derivatives thereof [13]. These compounds bind to exposed DNA fragments from cells that lost membrane integrity and successfully enabled preclinical necrosis imaging using positron emission tomography (PET) [14], single photon emission computed tomography (SPECT) [15], or magnetic resonance imaging (MRI) [16–18]. A particular class of NACAs on which we previously focused is cyanines, a class of organic compounds that are extensively used for their biocompatible fluorescent properties [19]. We demonstrated that some of these compounds exhibit excellent necrosis avidity by binding to cytoplasmic proteins that become available upon loss of membrane integrity [12, 20]. These cyanines enabled SPECT imaging of tumor necrosis when labeled with indium-111 [20–22].

**Figure 1:**
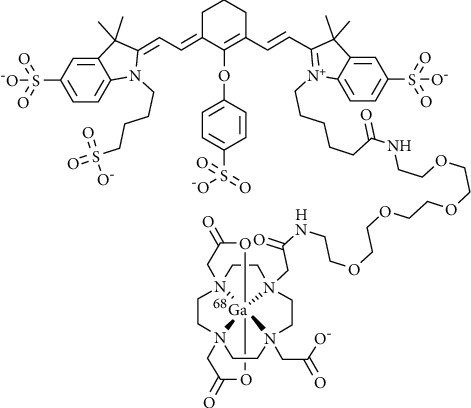
Chemical structure of [^68^Ga]Ga-DOTA-PEG4-IRDye800CW ([^68^Ga]Ga-1).

In our preliminary work, we labeled cyanine dye IRDye800CW with indium-111 for in vivo imaging of spontaneous tumor necrosis in a 4T1-Luc2 breast cancer model in mice [22]. Despite its rapid renal excretion, the indium-111-labeled IRDye800CW allowed the detection of tumor necrosis with high signal-to-noise ratios. Here, we demonstrate PET imaging of necrosis with [^68^Ga]Ga-DOTA-PEG_4_-IRDye800CW ([^68^Ga]Ga-1, Figure 1), a gallium-68-labeled IRDye800CW. Gallium-68 is a positron emitter that can be obtained from a germanium-68/gallium-68 generator, allowing hospital institutions to produce PET tracers without the requirement of an on-site cyclotron facility [23–26]. Moreover, PET has several advantages over SPECT imaging, such as higher sensitivity, shorter scan time, lower radiation exposure, more accurate quantification, and the possibility of dynamic image reconstruction [26, 27]. We hypothesize that the short half-life of gallium-68 (68 minutes) suits the fast pharmacokinetics of the IRDye800CW-based NACA better than that of the earlier described indium-111 [22]. This study is important for clinical translation as the lower amount of radioactivity required for PET imaging and the shorter half-life of gallium-68 will lead to lower radiation exposure from our necrosis probe.

## 2. Materials and Methods

### 2.1. Materials

Reagents were purchased from Sigma-Aldrich (St. Louis, MO, USA) unless stated otherwise. Solvents were purchased from Honeywell Riedel-de-Haën™ (Seelze, Germany). For [^68^Ga]GaCl_3_, a >1-year-old clinical-grade ^68^Ge/^68^Ga-generator eluting 500–550 MBq at time of use from Eckert and Ziegler (Berlin, Germany) was used. 4T1-Luc2 cells were purchased from PerkinElmer (Boston, MA, USA). Cell culture media was obtained from Sigma-Aldrich or Gibco Life Technologies (Waltham, MA, USA). HPLC-analyses were performed on a system from Waters (Milford, MA, USA) equipped with a sample manager FTN-R, a quaternary solvent manager-R, a 2998 PDA detector, a radiodetector consisting of a NaI crystal with a Canberra Osprey-DTB, and dedicated IFlow and Empower 3 software. HPLC method: Waters Symmetry C_18_ analytical column (4.6 × 250 mm, 5 *μ*m) and a gradient profile: 0-1 min 22% B, 1–20 min towards 51% B (solvent A: 0.2 M Tris-HCl buffer (pH 8.5) + 10% MeOH; solvent B: MeOH) at a flow rate of 1.0 mL/min. Dosis calibration was performed on an IBC dose calibrator with 30 mm lead shielding (Comecer, Joure, The Netherlands). Autoradiography was performed using super-resolution phosphor screens and a Cyclone® Plus Phosphor Imager (Perkin Elmer, Waltham, MA, USA). NIR fluorescence imaging was acquired on an Odyssey flatbed scanner system (800 nm channel, laser intensity 5.0; Li-Cor, Lincoln, NE, USA). In vivo imaging was carried out with VECTor^5^OI/CT (MILabs, Houten, The Netherlands). PET images were acquired with a high-energy general purpose 1.6 mm pinhole mouse collimator. Two-dimensional OI images were acquired with an integrated CCD camera system. The images were analyzed with Pi-Mod (version 3.901). The accumulated activity in the resected organs and tumors was accurately quantified on a Wizard 3″, 1480 *γ*-counter (PerkinElmer). Histochemical dead cell staining was performed using the DeadEnd™ Colorimetric Terminal deoxynucleotidyl transferase dUTP Nick end labeling (TUNEL) system (Promega, Madison, WI, USA) and subsequent imaging on a NanoZoomer 2.0HT digital slide scanner (Hamamatsu, Hamamatsu City, Japan).

### 2.2. Isolation [^68^Ga]GaCl_3_

[^68^Ga]GaCl_3_ was isolated as previously described [23]. Briefly, the ^68^Ge/^68^Ga-generator was preeluted with HCl (sterile and ultrapure, Eckert and Ziegler, 0.1 M, 6.0 mL, and 2.0 mL/min). After 2-3 h, the generator was eluted with HCl (0.1 M, 6.0 mL, and 2.0 mL/min), and the [^68^Ga]GaCl_3_ was trapped on a PEEK Bio-Safe column (2.1 Å–300 mm) including a 2 *μ*m filter frit (TrisKem), manually charged with PS-H+ cation exchange resin (Chromafix® PS cartridges, pore size 100 Å, particle size 100 *μ*m, 15 mg, preconditioned with 6 mL 0.1 M HCl). The column was dried by flushing with 10 mL air, and ^68^GaCl_3_ was collected by eluting with 100 *µ*L portions of NaCl (3 M, acidified with 0.05 M HCl, purified overnight from metal ions with Chelex 100 (Bio-Rad, CA, USA)). The concentration of the eluate (in MBq/*µ*L) was determined by a dose calibrator. The second 100 *µ*L portion contained the highest activity concentration.

### 2.3. Radiolabeling of [^68^Ga]Ga-DOTA-PEG_4_-IRDye800CW ([^68^Ga]Ga-1)

Precursor 1 was synthesized with high purity (>99%) as previously reported [22]. Into a conical vial, HEPES buffer (100 *µ*L, 1.5 M, pH 5.7, purified overnight from metal ions with Chelex 100) was added to 1 (4.8 *µ*g, 3.0 nmol). Then, freshly isolated [^68^Ga]GaCl_3_ (30 MBq) was added to 1, and the reaction vessel was sealed and heated at 90°C for 10 min. Then, the vial was cooled to room temperature over 5 min, and EDTA (4 mM, 5 *µ*L) was added to complex remaining free gallium. Radiochemical purity (RCP) of [^68^Ga]Ga-1 was determined by HPLC (*t*_*R*_ = 15.3 min). The reaction mixture was diluted with demineralized water to adjust osmolality to physiological concentration (0.154 M) [23]. Additionally, reference compound [^68^Ga]Ga-EDTA ([^68^Ga]Ga-2) was prepared by adding freshly isolated [^68^Ga]GaCl_3_ (30 MBq) to excess of EDTA (4 mM, 5 *µ*L) in HEPES buffer (100 *µ*L, 1.5 M, pH 5.7) without heating.

### 2.4. Preparation of Reference Compound Ga-DOTA-PEG_4_-IRDye800CW (Ga-1)

Into a conical vial, HEPES buffer (150 *µ*L, 1.5 M, pH 5.7, purified overnight from metal ions with Chelex 100) was added to 1 (72 *µ*g, 45 nmol). Then, gallium ICP standard (1000 mg/L Ga, 4.69 *µ*L, 67.25 nmol) was added, and the reaction vessel was sealed and heated at 90°C for 10 min. Then, the vial was cooled to room temperature over 5 min. HPLC analysis was performed of freshly prepared [^68^Ga]Ga-1, Ga-1, nonlabeled precursor 1, and a 1 : 1 mixture of Ga-1 and 1.

### 2.5. Determination of *n*-Octanol/PBS Distribution Coefficient (LogD_7.4_)

[^68^Ga]Ga-1 was labeled at a molar activity of 10 MBq/nmol, and 300 kBq was added *n*-octanol (500 *µ*L) and phosphate-buffered saline (PBS, 500 *µ*L). After vigorous vortexing, the vial was centrifuged at 16.1 × 1000 g for 3 min. The n-octanol phase was carefully pipetted into a new vial, and both layers were centrifuged again. From both the organic and the aqueous layers, samples (10 *µ*L) were taken out and counted for activity on a *γ*-counter. LogD values were calculated as follows: Log ([NACA]_*n*-octanol_/[NACA]_PBS_). This experiment was performed in triplicate.

### 2.6. In Vitro Dead/Alive Cell Uptake Assay

4T1-Luc2 cells were cultured in the RPMI-1640 medium containing 10% fetal bovine, penicillin (100 U/mL), and streptomycin (100 mg/mL) at 37°C under a humidified atmosphere with 5% CO_2_. The cells were seeded in four 24-well plates (1.0 × 10^5^ cells per well) and grown until fully confluent. Then, the medium was removed from half of the wells from every plate, and the cells were killed with ethanol (50 *µ*L, 70%) and washed once with PBS. The live cells in the other half of the wells were washed with PBS. Subsequently, all cells were incubated for 30 min with or without [^68^Ga]Ga-1 (6.7 MBq/nmol; 0.67 MBq/mL, equivalent to 100 nM in culturing medium) or [^68^Ga]Ga-2 (0.67 MBq/mL in culturing medium). The cells of two plates were washed thrice with PBS, collected with NaOH (1 mL, 1.0 M), and transferred to tubes for accurate *γ*-counting. The cells of the other two plates were washed thrice with PBS and stored at −20°C overnight for radioactive decay. The following day, fluorescence uptake was determined by imaging the whole plate on an Odyssey. This experiment was performed in *n* = 6.

### 2.7. Animals

Four female BALB/cAnNRj-nude mice (6–8 weeks old) were housed in ventilated cages in groups of four mice and were provided standard laboratory animal food pellets and water ad libitum. A week after arrival, 1.0 × 10^4^ 4T1-Luc2 cells suspended in 30 *µ*L Matrigel/PBS (1 : 1) were injected bilaterally on the shoulders. Tumor growth was monitored three days per week with a caliper.

### 2.8. PET/OI/CT Imaging

Four tumor-bearing mice received an intravenous injection of freshly prepared [^68^Ga]Ga-1 (6.4–9.0 MBq, 2 nmol, 200 *µ*L) in the tail vein. Imaging was performed on a VECTor^5^OI/CT at 1 h postinjection (p. i.) under isoflurane anesthesia (4% induction, 1.5–2% maintenance in 100% O_2_), whilst maintaining the body temperature constant. First, 2D near infrared-fluorescence imaging (NIR-FLI) was performed with a 732 nm excitation and 775 nm emission filter for 400 ms with a 4 × 4 binning. Then, the mice received an intraperitoneal injection of D-luciferin (150 mg/kg) for 2D bioluminescence imaging (BLI) of the 4T1-Luc2 cells, without moving the mouse in the bed. During the onset of the BLI signal, PET images of the 5.0 cm axial field of view were obtained over a total scan time of 30 min in list mode, followed by a 2 min full-body CT. Finally, 2D BLI was performed with an open filter for 400 ms, with a 4 × 4 binning. Acquired PET images were reconstructed using MLEM with 128 iterations, 1 subset on a 36 × 36 × 35 mm matrix with 0.8 × 0.8 mm isotropic voxels, and a decay correction of 68 min was applied. OI images were processed using ImageJ.

### 2.9. Ex Vivo Analysis

After imaging, blood, tumors, and other organs were collected, weighted, and activity was accurately quantified by *γ*-counting. After counting, two tumors were sliced in half and placed on a photosensitive plate for detection of radioactivity. The tumor halves were stored at −20°C until the activity decayed, after which NIR fluorescence was detected on an Odyssey. The remaining tumors were frozen in liquid nitrogen directly after resection. Adjacent 10 *µ*m cryosections were prepared for NIR fluorescence imaging and TUNEL staining.

### 2.10. Statistics

All data are expressed as the mean ± standard deviation (SD). Outliers were identified and excluded by a *q*-test. Significance was determined with unpaired two-tailed *t*-tests using Microsoft Excel for Mac 2016.

## 3. Results

### 3.1. Radiolabeling

[^68^Ga]GaCl_3_ was isolated in 100 *µ*L fractions with a concentration of 1.13–1.67 MBq/*µ*L. After purification of the activity, precursor 1 was reliably radiolabeled at a molar activity of 10 MBq/nmol. The radiochemical purity (RCP) of [^68^Ga]Ga-1 was analyzed by radio-HPLC and was 97.2 ± 0.9% (*n* = 4, Supplementary data 1). Unreacted ions were chelated by EDTA, which was added at the end of the reaction. The [^68^Ga]Ga-EDTA eluted at 2 min on our earlier reported HPLC method [22]. Further instrumental analysis confirmed that this critical method separates Ga-1 from nonlabeled precursor 1, while [^68^Ga]Ga-1 and Ga-1 coelute (Supplementary data 2). Interestingly, the gallium-labeled material eluted later than the unlabeled precursor, whereas the indium-labeled counterpart was reported to elute earlier [22]. The NACA retained an RCP of >94% after an hour at room temperature. It must be noted that all experiments were started within 30 min after the start of synthesis (Supplementary data 3). The hydrophilicity at physiological pH (LogD_7.4_) was determined by the separation of [^68^Ga]Ga-1 between *n*-octanol and PBS. [^68^Ga]Ga-1 was highly hydrophilic with a LogD_7.4_ of −3.77 ± 0.37.

### 3.2. In Vitro Dead Cell Binding

**Figure 2:**
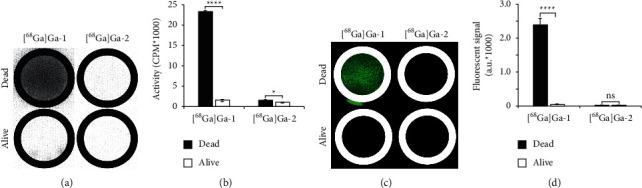
In vitro dead cell binding of [68Ga]Ga-DOTA-PEG4-IRDye800CW ([68Ga]Ga-1) and [68Ga]Ga-EDTA ([68Ga]Ga-2). (a, c) Dead or alive 4T1-Luc2 cells in a 12-well plate, treated with either [68Ga]Ga-1 or [68Ga]Ga-2. In (a), the autoradiography signal and in (c), the corresponding 800 nm channel are represented in green. In (b), the quantification of bound radioactivity and in (d), the quantification of fluorescent signal are depicted. Error bars represent standard error of the mean. ns, no significant difference. ^*∗*^*P* < 0.05., ^*∗∗∗∗*^*P* < 0.0001.

Figure 2 shows the in vitro dead cell binding of [^68^Ga]Ga-1 and [^68^Ga]Ga-2, as a negative control. Although there is a difference in binding to dead cells as compared to alive cells by [^68^Ga]Ga-2 (*P*=0.0195), the difference for [^68^Ga]Ga-1 is considerable (*P*=1.50 × 10^−7^). Moreover, no autofluorescence is observed from [^68^Ga]Ga-2-treated cells (*P*=0.98, Figures 2(c) and 2(d)). As well the fluorescence signal of [^68^Ga]Ga-1 revealed significant binding to dead over alive cells (*P*=2.45 × 10^−11^).

### 3.3. In Vivo Imaging

**Figure 3:**
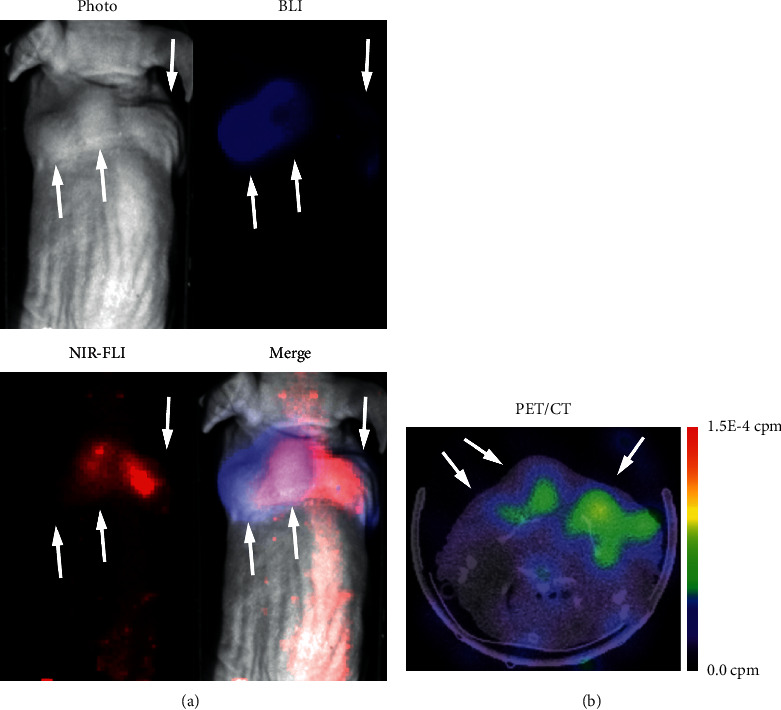
Representative in vivo images of 4T1-Luc2 tumor-bearing mouse 1 at 1 h postinjection with [68Ga]Ga-1. (a) BLI/NIR-FLI imaging: BLI signal in blue and NIR-FLI signal in red. (b) PET/CT image mouse. The white arrows indicate the location of tumors, and the dashed white line in the merged picture of (a) indicates the approximate level of the transverse slice in (b).

The mice were inoculated with luciferase-expressing 4T1 cells, which enabled BLI of viable tumor regions upon injection of D-luciferin. The images were obtained as 2D BLI images, and the spontaneously developed necrotic core appeared thereby as a “dark” spot in the BLI image and could be detected using the NIR-fluorescence properties of [^68^Ga]Ga-1 (Figure 3(a)). Furthermore, the tumors were visible in PET images, and the tumoral PET signal corresponds well with the localization of the NIR-FLI signal (Figure 3(b)). The tumor on the left shoulder grew in two lumps and demonstrated a relatively high BLI signal, while the tumor on the right demonstrated less BLI signal. Moreover, higher FLI and PET signals in the right tumor confirmed a higher presence of tumor necrosis. Optical imaging of the other three mice is presented in Supplementary data 4.

### 3.4. Ex Vivo Analysis

**Figure 4:**
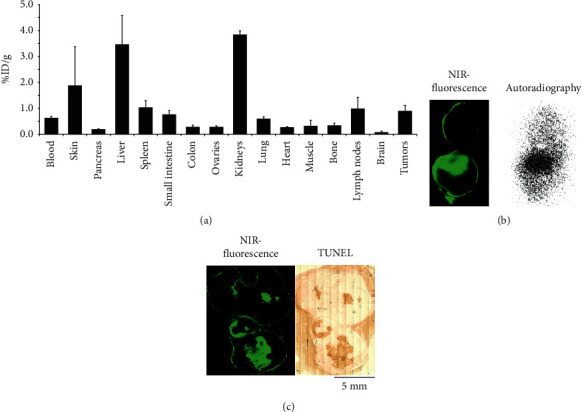
Biodistribution and ex vivo analysis of tumor-bearing mice injected with [68Ga]Ga-1. (a) Biodistribution of [68Ga]Ga-1 in 4T1-Luc2 tumor-bearing mice at 1.5 h postinjection; error bars indicate standard deviation, n = 4 (for tumors n = 8). (b) Fluorescence and radioactivity uptake in the dichotomized left tumor from a [68Ga]Ga-1-treated mouse 2. (c) Fluorescence uptake and TUNEL dead cell staining in a 10 µm frozen tumor section from [68Ga]Ga-1-treated mouse 4.

The mice were sacrificed after imaging, and a biodistribution was performed for accurate quantification of the tracer uptake in the selected organs and tumors (Figure 4(a)). The organs that stand out are the excretion organs kidneys and liver. Tissues with rapidly dividing cells (skin, intestines, and lymph nodes) also showed high uptake of [^68^Ga]Ga-1. The average tumoral radioactivity uptake was 0.89 ± 0.24% ID/g (*n* = 8) at 1 h postinjection, which is slightly higher than the activity left in blood (0.61 ± 0.08% ID/g, *n* = 4). In the dichotomized tumor, a heterogenous radioactivity and fluorescence uptake pattern was observed. This colocalization confirmed the heterogenous uptake of the dual-modal contrast agent. Subsequently, the fluorescence uptake colocalized with the histopathological dead cell staining, as demonstrated in the frozen tissue sections (Figures 4(b) and 4(c)). Due to the short half-life of gallium-68, it was practically impossible to perform autoradiography on a frozen tissue section. Colocalisation of both radioactivity and TUNEL dead cell staining with the fluorescence uptake revealed specific binding to the dying regions of the tumor by the dual-modal contrast agent. Additional ex vivo analysis is presented in Supplementary data 5.

## 4. Discussion

In our previous work, we performed SPECT-imaging of necrosis using indium-111 labeled IRDye800CW, as a necrosis-avid contrast agent (NACA). This NACA was retained in the necrotic cores of 4T1-Luc2 tumors for several days, enabling SPECT imaging of tumor necrosis with high contrast [22]. However, we observed rapid clearance of the tracer from the body, which is most likely caused by its high hydrophilicity (LogD_7.4_: −3.77 ± 0.37). Typically, tracers with fast kinetics are paired with radionuclides with short half-lives. The in vivo kinetics of IRDye800CW did therefore not match the relatively long half-life of indium-111 [28]. Hence, we proposed to label the NACA with a short-lived isotope.

As previously mentioned, the higher sensitivity of PET imaging combined with the use of short-lived radioisotopes results in considerably lower radiation exposure to the patient as compared to SPECT [26]. The generator-produced PET isotope gallium-68 has the advantage that it can be produced in sites without a cyclotron infrastructure, making gallium-68 an accessible radioisotope. Moreover, gallium-68 can directly be incorporated in the DOTA-chelator of DOTA-PEG_4_-IRDye800CW. However, there is a resolution discrepancy between preclinical and clinical PET and SPECT imaging. Clinical PET imaging is less hampered by the range that positrons travel through soft tissue due to the larger size of humans over laboratory animals. The introduction of multipinhole collimators, on the other hand, drastically improved the imaging quality of preclinical SPECT, but this technique is not (yet) available for clinical SPECT cameras [29].

The luciferase-expressing tumor cells facilitated bioluminescence imaging of living cells in the tumor [30]. The order in which the different imaging modalities were recorded was important to exclude potential interference of bioluminescence with NIR-FLI imaging. Hence, NIR-FLI imaging was performed before D-luciferin injection. We ensured that the ip injection was performed without moving the mouse in the bed. The bioluminescence signal has a gradual onset and peaks at around 20 minutes postinjection of the substrate [30], during which PET/CT imaging was performed. After the PET/CT imaging sequence, BLI imaging was performed. By keeping the positioning of the mouse constant throughout the imaging sequence, an overlay could be created of the NIR-FLI and BLI images.

The overlay of NIR-FLI with BLI imaging (Figure 3(a)) and colocalization of TUNEL dead cell staining and autoradiography signal with NIR fluorescence in the dissected tumors confirmed specific uptake of the tracer by the necrotic tumor core (Figures 4(b) and 4(c)). The heterogenous intratumoral uptake pattern of [^68^Ga]Ga-1 is identical as observed with the earlier described [^111^In]In-1 [22]. The uptake of [^68^Ga]Ga-1 by the tumor necrosis was also observed on PET images. Due to the early time point after injection, which is necessary for gallium-68, the background of the PET signal was relatively high. The nuclear imaging setup used in this study is originally designed for SPECT imaging and is not an optimal setup for PET imaging [31]. The tumor uptake of [^68^Ga]Ga-1 was 0.89 ± 0.24% ID/g, and a tumor-to-blood ratio (TBR) of 1.44 ± 0.23 was based on the biodistribution data. When compared with other gallium-68-labeled NACAs in the literature, this uptake and TBR would yield higher quality PET images on more dedicated PET cameras [14, 32, 33].

The main limitation of the presented study is that the collimator imager has limited sensitivity. A dedicated coincidence PET scanned will be more sensitive and will provide better imaging quality. Nonetheless, does the VECTor^5^OI/CT serve as an appropriate setup for the proof of concept of PET imaging with [^68^Ga]Ga-1. Furthermore, only a single subcutaneous mouse model is demonstrated. 4T1-Luc2 cells grow as rapidly proliferating tumors, which spontaneously develop necrotic cores, and have been a useful model for tumor necrosis in previous works. Another limitation is that quantification of necrosis on the tumor sections does not represent the overall necrosis in the tumors, and therefore, direct correlation of tumor necrosis to our probe is difficult. However, we can see a correlation between the inherent fluorescence of the probes with TUNEL staining. At 1 h postinjection, the background signal is still relatively high, especially in the liver and kidneys. This may limit the translational application for tumors located in these organs.

Nevertheless, our study showed that tumor necrosis can be detected early after injection of [^68^Ga]Ga-1. PET imaging with this tracer will result in a considerably lower radioactivity exposure than injection with the earlier described counterpart, labeled with indium-111 [22]. The low toxicity of IRDye800CW makes it an interesting starting point for developing necrosis avid contrast agents with strong translational power [34]. We demonstrated necrosis binding in a model with spontaneously developing tumor necrosis; in future projects, we will explore the detection of therapy-induced tumor necrosis.

To conclude, we explored PET imaging of spontaneously developing tumor necrosis with [^68^Ga]Ga-1 as a proof of concept. [^68^Ga]Ga-1 enabled PET imaging of spontaneous necrotic tumors. Moreover, in vitro dead cell binding and ex vivo binding profile elucidated specific binding of the NACA to dead tumor cells. Our study may pave the way towards the development of cyanine-based PET tracers for clinical necrosis imaging.

## Data Availability

The data used to support the findings of this study are available from the corresponding author upon request.

## Supplementary Materials

Supplementary Materials Supplementary materials 1–3 contain (radio-) HPLC analysis of [^68^Ga]Ga-1, including stability tests and HPLC-method verification. Supplementary material 4 contains in vivo imaging of the remaining three mice used in this study. Supplementary data 5 contains further ex vivo analysis on resected necrotic tumors.
