# Supplementary material for: In Vivo Evaluation of Gallium-68-Labeled IRDye800CW as a Necrosis Avid Contrast Agent in Solid Tumors
Source: Contrast Media Mol Imaging. 2021 Dec 13;2021:2853522. doi: 10.1155/2021/2853522 (PMC8687856; doi:10.1155/2021/2853522)
Supplement: Supplementary Materials — 1–3 contain (radio-) HPLC analysis of [68Ga]Ga-1, including stability tests and HPLC-method verification. Supplementary material 4 contains in vivo imaging of the remaining three mice used in this study. Supplementary data 5 contains further ex vivo analysis on resected necrotic tumors. [file 2853522.f1.docx]

# Supplementary data


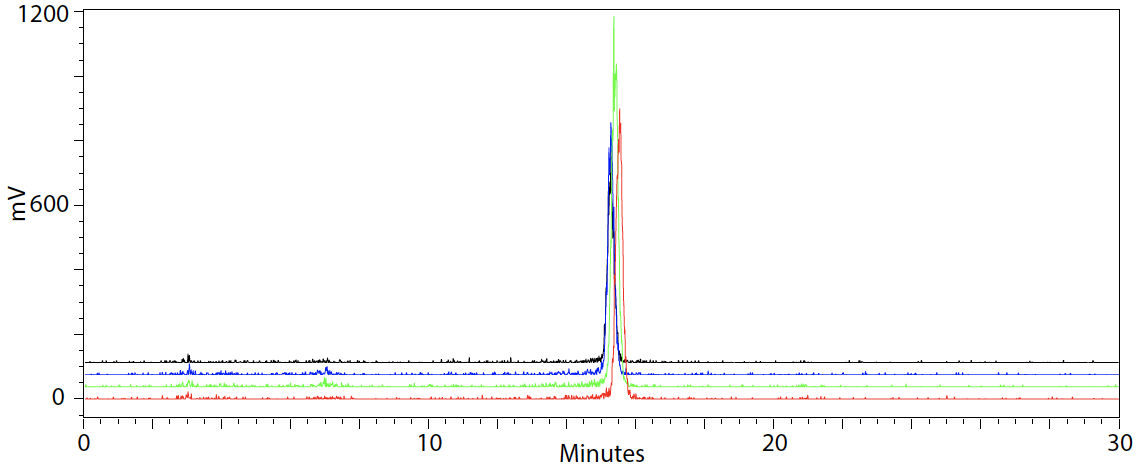


Supplement 1: Radio-HPLC analysis, overlay of four QC chromatograms of [^68^Ga]Ga-**1** (10 MBq/nmol) for *in vivo* studies. Average RCP: 97.2±0.9%.

Supplement 2: HPLC separation precursor **1** and Ga-**1**. In black: radio-HPLC chromatogram of [^68^Ga]**1**; in blue: HPLC chromatogram of Ga-**1**, in green: HPLC chromatogram of Ga-**1** spiked with precursor **1**; in red: HPLC chromatogram of precursor **1**.


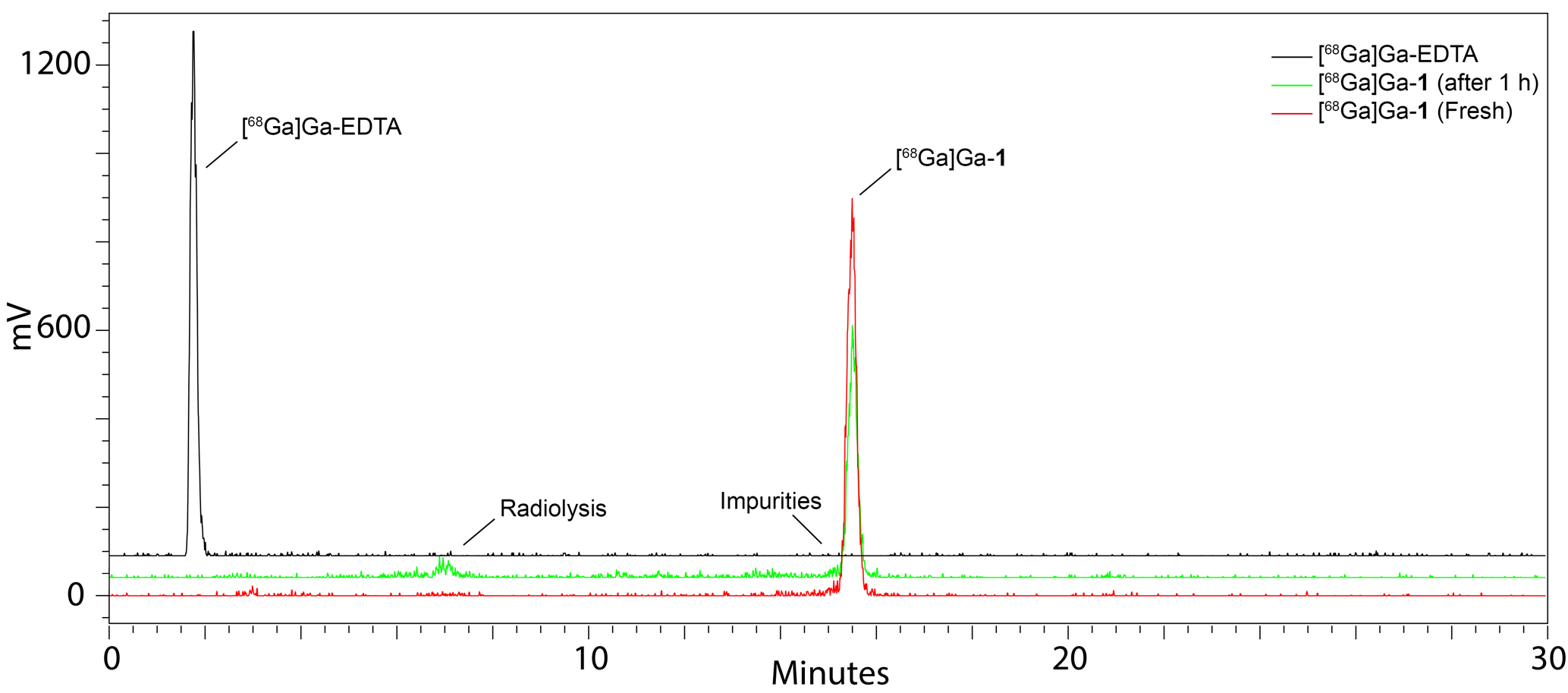


Supplement 3: Radio-HPLC analysis, overlay of three chromatograms. In red: freshly prepared [^68^Ga]Ga-DOTA-PEG_4_-IRDye800CW ([^68^Ga]Ga-**1**, 10 MBq/nmol, RCP: 98.2%); in green: [^68^Ga]Ga-**1** after one hour at room temperature (RCP: 94.9%); in black: [^68^Ga]Ga-EDTA.


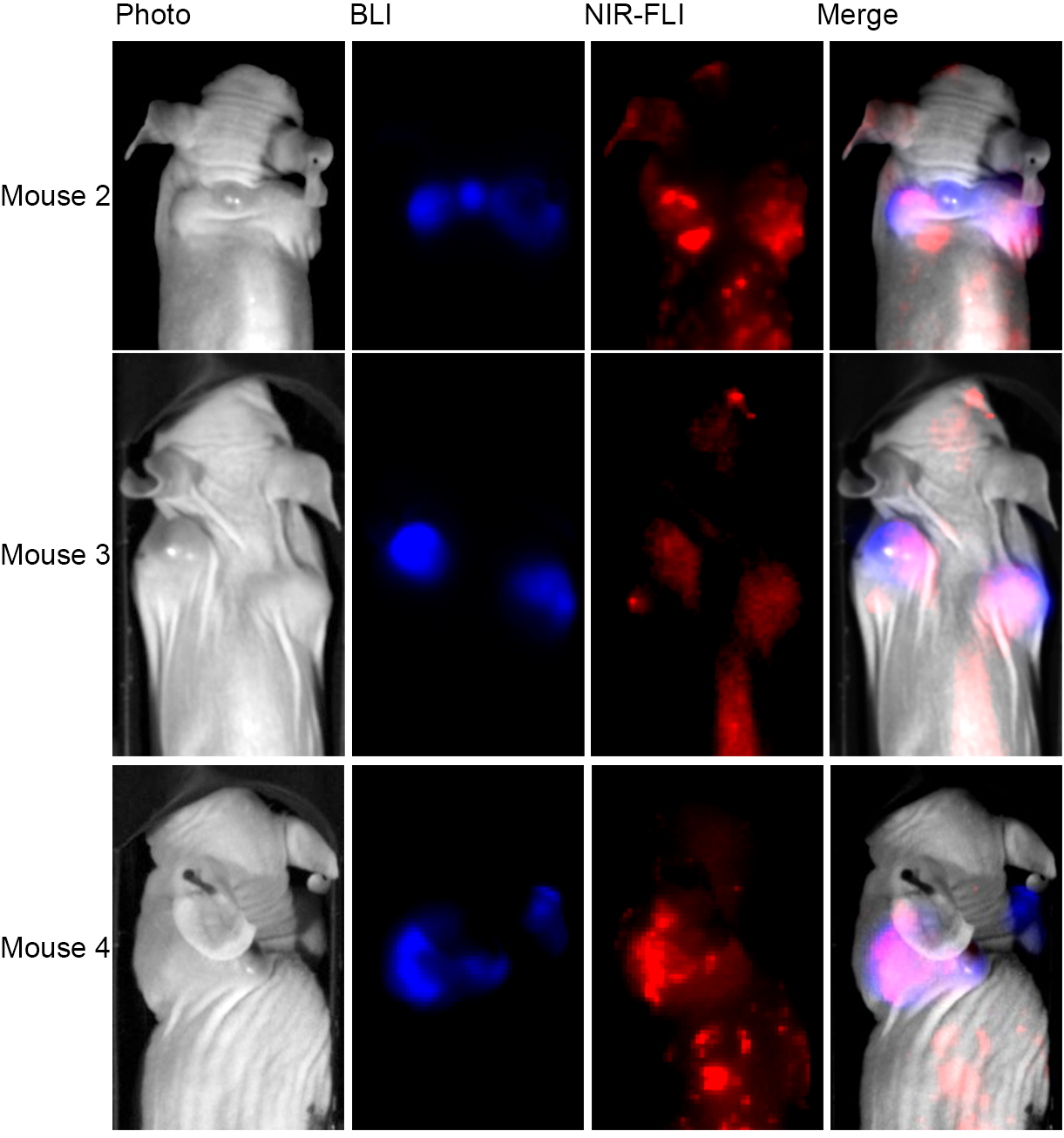


Supplement 4: FLI and BLI images of the other three mice. The mice got contaminated by excreted fluorescent contrast agent in the cage after injection. The left tumor of mouse 3 was used for Figure 4**B** and the left tumor of mouse 4 was used for Figure 4**C**.


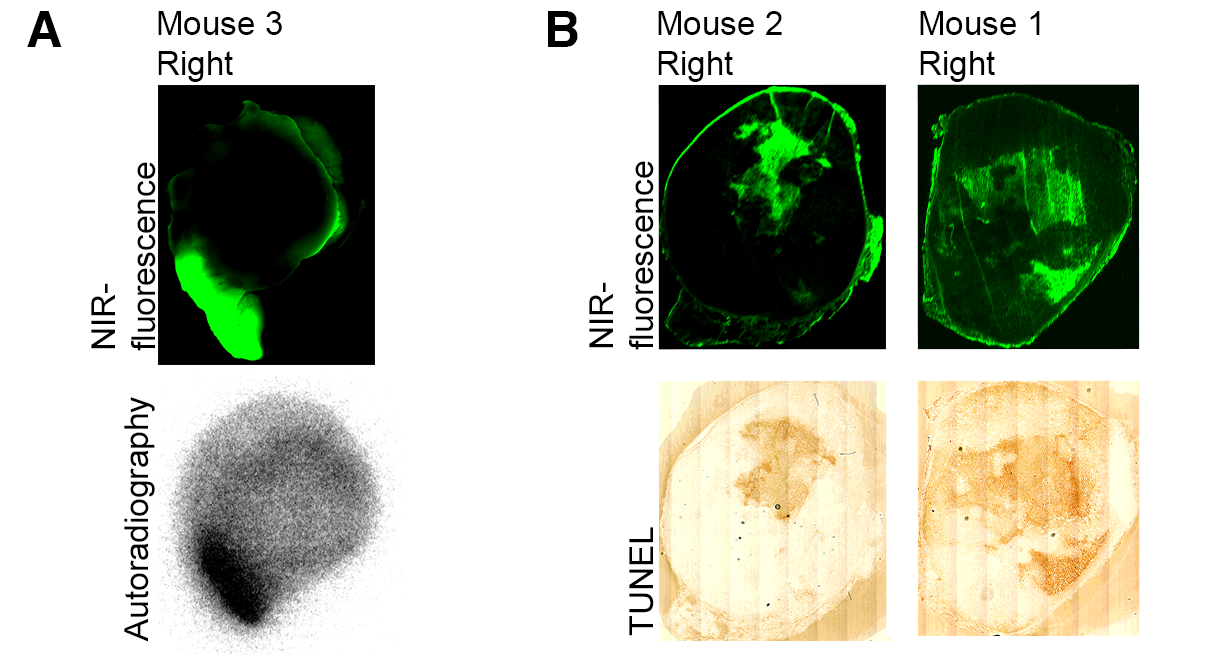


Supplement 5: Extra *ex vivo* analysis. **A**: dichotomized right tumor from mouse 3, analyzed with autoradiography and NIR-fluorescence imaging. **B**: Frozen tumor sections from mouse 1 and mouse 2 analyzed by NIR-fluorescence imaging and TUNEL dead cell staining.
